# Defensin Interactions in Relation to Monoclonal and Disease-Related Proteinase 3 Antibodies Binding at the Catalytic Site

**DOI:** 10.3390/antib12010023

**Published:** 2023-03-13

**Authors:** Morten Zoega, Nicole Hartwig Trier, Rikke Guldhammer Nejrup, Anna Chailyan, Tina Friis, Peter Højrup, Gunnar Houen

**Affiliations:** 1 Department of Autoimmunology and Biomarkers, Statens Serum Institut, DK-2300 Copenhagen S, Denmark; 2 Department of Biochemistry and Molecular Biology, University of Southern Denmark, DK-5230 Odense M, Denmarkphp@bmb.sdu.dk (P.H.)

**Keywords:** anti-neutrophil cytoplasm antibody (ANCA), catalytic site, epitopes, monoclonal antibodies, proteinase 3

## Abstract

Proteinase 3 (PR3) is a neutrophil granulocyte enzyme and an autoantigen found in several forms of vasculitis. Due to the diagnostic and clinical importance of antibodies (Abs) to PR3, it is important to characterize the protein and the nature of its epitopes. Here, we have characterized PR3 monoclonal antibodies (MAbs) and disease-associated Abs and their dependency on the PR3 structure and modifications, especially interactions with α-defensins. Three MAbs (HYB 172-01, 172-04, 172-05), which bind to PR3 in its native and denatured forms and provide the disulphide bridges, were intact. α-1-antitrypsin (AT) binds to purified human neutrophil granulocyte PR3 and inhibits its proteolytic activity, towards a small synthetic peptide substrate and a large protein substrate (casein). AT also inhibited the binding of the three MAbs to PR3, indicating that they bind in a region affected by AT binding. However, the MAbs did not inhibit PR3 proteolytic activity with a small substrate, showing that they bound at the active site without restricting access to the substrate cleft. Patient-derived Abs showed essentially the same characteristics as the MAbs, with important implications for vasculitis diagnostics and pathophysiology. Current findings illustrate that PR3 epitopes depend on the three-dimensional structure of the PR3/defensin complex, and that the epitopes depend to a smaller or larger degree on PR3/defensin associations.

## 1. Introduction

Proteinase 3 (PR3) is a serine protease and a constituent of neutrophil granulocyte α-granules, where it is present together with myeloperoxidase (MPO) and several other antibacterial proteins and peptides, including the α-defensins [1,2]. PR3 is also an autoantigen found in various forms of vasculitis [3,4,5,6]. Molecular interaction studies, e.g., using epitope mapping, may shed some light on autoimmune vasculitis and, therefore, the major focus has been on elucidating the immunogenicity and antigenicity of PR3 in relation to diagnostics and pathophysiology of vasculitis [6,7,8,9]. PR3 is encoded by a single gene located together with two other elastase-like genes at a single genetic locus, and it is synthesized as a pre-pro-protease, which is processed to mature PR3 by proteolytic removal of a signal peptide, an N-terminal pro-dipeptide and a C-terminal pro-peptide [2,10,11,12,13]. The structure of PR3 is similar to that of other serine proteases, e.g., elastase, and it is inhibited by α1-antitrypsin (AT), with which it forms complexes similar to elastase [2,8,10,11,14,15,16,17].

The location and nature of antibody (Ab) epitopes on PR3 have been studied extensively using sera from patients with ANCA (anti-neutrophil cytoplasm antibody) vasculitis and monoclonal antibodies (MAbs), in combination with purified native mature PR3, recombinant PR3 constructs and synthetic peptides. Most Abs react with conformational epitopes and depend on intact disulfide bridges of PR3 [9,10,18]. This may partly explain why many studies have yielded different results. In general, PR3 expressed in *E. coli* has shown no or only weak binding of patients’ autoantibodies (AuAbs), presumably due to incorrect folding. However, PR3 expressed in eukaryotic/mammalian expression systems has shown binding of Abs from some but not all patients and binding depended for a subset on removal of the pro-dipeptide and correct glycosylation [9,19,20,21,22,23,24,25,26,27,28,29]. Studies with synthetic peptides and peptides from proteolytic digestion of PR3 have yielded some detailed molecular information on epitopes for patient PR3 Abs, pointing to the involvement of regions around the catalytic site [18,30,31,32,33]. MAbs against PR3 have been used for epitope mapping, which has confirmed the importance of correct folding in regions around the catalytic site for reactivity [23,27,28,29,34,35,36,37,38].

We have previously characterized the glycosylation of native PR3 from neutrophil granulocytes and shown that it associates strongly with several α-defensins by a combination of non-covalent and covalent interactions [38]. In light of this knowledge of PR3 modifications and defensin associations, we have undertaken a characterization of the interaction of PR3 with several MAbs and AuAbs from human sera. Here, we show that PR3 Abs bind to conformational epitopes located at the active catalytic site and that the behavior of PR3 in immunoassays is strongly influenced by associated defensins.

## 2. Materials and Methods

### 2.1. Reagents

Mouse MAbs against PR3 (HYB 172-04/IgG2a,κ (clone 4A3), HYB 172-05/IgG2a,κ (clone 4A5), HYB 172-01/IgG1,κ (clone 6A6), HYB 172-03/IgG1,κ (clone 4F9) and HYB 206-1/IgG1,κ (clone 11E2), as well as mouse MAbs to serum amyloid P (SAP) HYB 281-05/IgG1,κ, (clone 14B4), mannan-binding lectin (MBL) HYB 131-01/IgG1,κ, (clone 3B6) and GC-globulin HYB 249-10/IgG1,κ (clone 4B9) and rabbit PR3 antiserum were produced and tested in-house using conventional technology [34,39]. The 172 series of MAbs are identical to previously described clones (HYB 172-01–HYB 172-05) produced by the immunization of mice with a Triton X-100 extract of purified neutrophil granulocyte α-granules and screening of clones using purified PR3 [34]. Mouse PR3 MAbs 1B10 and 2E1 were from VWR/Avantor (Copenhagen, Denmark) and alkaline phosphatase (AP)-conjugated goat Abs against mouse IgG and rabbit IgG were from Sigma (St. Louis, MI, USA). For detection of non-specific binding and as negative controls, the following Abs were used: rabbit Igs; mouse IgG1; and mouse IgG2a (DAKO, Copenhagen, Denmark). Other reagents were from the following suppliers: PR3 (Wieslab/Eurodiagnostica, Lund, Sweden and Arotec, Wellington, New Zealand); human α1-antitrypsin (Calbiochem, Merck, Germany); phenylmethanesulfonyl fluoride (PMSF) (Roche, Basel, Switzerland); MeOSuc-Ala-Ala-Pro-Val-OH; MeOSuc-Ala-Ala-Pro-Val-CMK; MeOSuc-Ala-Ala-Pro-Val-pNA (Bachem, Weil am Rhein, Germany); ovalbumin, BCIP/NBT (5-Bromo-4-chloro-3-indolyl phosphate/Nitro blue tetrazolium) substrate tablets, dithiothreitol (DTT), *para*-nitrophenylphosphate (*p*-NPP) substrate tablets, Triton X-100, Triton X-114, dimethylsulfoxide (DMSO) (Sigma, St. Louis, MI, USA). Furthermore, 20-mer peptides with 10 amino acid overlap were synthesized by Schäfer-N (Copenhagen, Denmark). An extra cysteine was introduced in the N-terminal end of all 20-mer peptides as a tag for covalent coupling of the PR3 peptides to NH-activated microtitre plates. The coating buffer (0.015 M Na_2_CO_3_, 0.035 M NaHCO_3_, 0.02% (*w*/*v*) NaN_3_, pH 9.6), incubation buffer (0.04 M Tris-HCl, 0.01 M Tris-base, 0.15 M NaCl, 0.05% (*v*/*v*) Tween 20, 0.02% (*w*/*v*) NaN_3_, 0.2% (*w*/*v*) bovine serum albumin, pH 7.5) and TTN buffer (0.05 M Tris-HCl, 0.3 M NaCl, 1% (*v*/*v*) Tween 20, pH 7.5) were from SSI Diagnostica (Hillerød, Denmark). Additionally, 1.0 M ethanolamine-HCl was from GE Healthcare (Uppsala, Sweden). Human serum samples from healthy controls, from persons tested for ANCA and from patients with a diagnosis of granulomatosis with polyangiitis (GPA) were from Biobanks at SSI. Patients and controls consented to the use of their samples for research and all samples were used anonymously, therefore waiving the necessity of ethical committee consent.

### 2.2. PR3 Purification

SSI PR3 was purified as previously described [38,40,41]. Briefly, PR3 from α (azurophilic) granules initially isolated from human neutrophil granulocytes was purified by extraction with Triton X-114. Temperature-induced separation, ion-exchange chromatography, purity and cross-reactivity with other human neutrophil granule proteins were evaluated by sodium dodecyl sulfate-polyacrylamide gel electrophoresis (SDS-PAGE) in combination with silver staining, western blotting and enzyme-linked immunosorbent assay (ELISA) using a combination of Abs against PR3 and other granulocyte proteins (elastase, MPO, cathepsin, azurocidin, lactoferrin and lysozyme).

### 2.3. SDS-PAGE

SDS-PAGE was performed as previously described [38]. Briefly, 1 µg of purified PR3 was mixed with a sample buffer with or without a reducing agent, boiled for one min and applied to 4–20% Tris–glycine SDS-PAGE gels (Invitrogen, Carlsbad, CA, USA). The following visualization was either carried out by colloidal Coomassie Brilliant Blue staining or mass spectrometry-compatible silver staining [42].

### 2.4. Western Blotting

PR3 was electro-blotted as previously described [38]. Briefly, PR3 from SDS-PAGE gels was transferred to polyvinyldifluoride (PVDF) membranes using an iBlot Dry blotting system according to the instructions of the manufacturer (Invitrogen, Carlsbad, CA, USA). After electro-transfer, the nitrocellulose membrane was blocked in an TTN buffer. Lanes were incubated with PR3 Abs and Abs to other neutrophil granule proteins (elastase, MPO, cathepsin, azurocidin, lactoferrin and lysozyme) diluted according to in-house determined dilutions. Secondary AP-conjugated Abs (Goat anti-mouse IgG or Goat anti-rabbit IgG) were incubated with the respective lanes for 1 h. The bound Abs were visualized with BCIP/NBT and development was stopped by washing them in water.

### 2.5. PR3 ELISA

PR3 Abs were detected by ELISA with the PR3 antigen coated in wells of microtitre plates. Briefly, microtitre plates (Maxisorp, Nunc, Roskilde, Denmark) were coated overnight at 4 °C with 100 μL/well of human PR3 in coating buffer (1 μg/mL) in the presence or absence of 0.01% Triton X-100. After washing and blocking with an incubation buffer, the Abs were diluted in incubation buffer according to the manufacturer’s instructions and incubated for 1 h at ambient temperature. The plates were washed three times and incubated for 1 h at ambient temperature with AP-conjugated secondary Abs diluted 1:3000 in an incubation buffer. *p*NPP was used as a substrate and the absorbances were measured at 405 nm after 30 min incubation at ambient temperature with background subtraction at 650 nm. In some experiments, various amounts of inhibitors were pre-incubated with PR3 before the direct coating of the inhibitor-PR3 complex in the wells of microtitre plates. In other experiments, various amounts of inhibitors were added to wells together with Abs directly or after 1 h of pre-incubation.

### 2.6. PR3 Capture ELISA

PR3 Abs were detected by capture ELISA, with the PR3 presented by immobilised MAbs to PR3. Briefly, microtitre plates (Maxisorp, Nunc, Roskilde, Denmark) were coated overnight at 4 °C with PR3 MAbs (1 or 0.05 μg/mL) in a coating buffer. After washing and blocking with an incubation buffer, purified PR3 (2 ng/well) was diluted in an incubation buffer and incubated for 1 h at an ambient temperature. The plates were washed three times and incubated for 1 h at an ambient temperature with PR3 polyclonal Ab (PAb) (1:1000) or GPA patient sera (1:1000) for 1 h at an ambient temperature. The plates were washed three times and incubated for 1 h at an ambient temperature with AP-conjugated secondary Ab (goat anti-rabbit IgG or goat anti-human IgG) diluted 1:3000 in an incubation buffer. *p*-NPP was used as a substrate and the absorbances were measured at 405 nm with background subtraction at 650 nm after 30 min of incubation at an ambient temperature.

### 2.7. PR3 Proteolytic Activity and Inhibition of Antibody-Antigen Binding

Substrate (MeOSuc-Ala-Ala-Pro-Val-OH) and low molecular weight inhibitors (PMSF, MeOSuc-Ala-Ala-Pro-Val-CMK) were dissolved in DMSO (25 mg/mL) and diluted in an assay buffer (PBS, 0.1% Tween 20) to 0.3 mg/mL and 0.75 mg/mL, respectively, whereafter PR3 was added to 20 µg/mL and the absorbance measured at 405 nm. The same concentrations of substrate and inhibitor were used in ELISA inhibition experiments, where Abs were used as described under ELISA and human α1-antitrypsin or ovalbumin was added in the concentrations indicated in figures and tables.

### 2.8. Peptide CovaLink-NH ELISA

Synthetic 20-mer peptides from the sequence of fully processed mature and active PR3 [10,11,12] were coupled to activated CovaLink-NH ELISA microtitre plates (Nunc, Roskilde, Denmark) via the cysteine added to the N-terminus of the synthetic 20-mer peptides following the instructions of the manufacturer. Patient sera diluted 1:100 in an incubation buffer were added and incubated 1 h at an ambient temperature. Bound Abs were detected as described under ELISA.

### 2.9. Surface Plasmon Resonance (SPR) Assays

The binding of PR3 MAbs to PR3 was investigated by SPR using the BIAcore system (GE Healthcare, Uppsala, Sweden). The binding measurements were carried out using a Biacore 1000 instrument equipped with a CM5 sensor chip (research grade chip). The HBS-‘E buffer pH 7.4 (10 mM 4-[2-hydroxyethyl] piperazine-1-ethane-sulfonic acid (HEPES), 3.0 mM EDTA, 150 mM NaCl) was used as the running buffer. PR3 was immobilized to the carboxy-methylated dextran sensor surface by amine coupling according to the manufacturer’s instructions. Briefly, the CM5 chip surface was activated with an 8 min injection (10 µL/min) of a 1:1 (*v*/*v*) mixture of 0.1 M NHS (N-Hydroxysuccinimide) and 0.4 M EDC (1-Ethyl-3-(3-dimethylaminopropyl)-carbodiimide hydrochloride), followed by an 8 min injection (10 µL/min) of PR3 (0.5 mg/mL in 10 mM acetate buffer, pH 4.0), resulting in an immobilization level of 1287 RU. The remaining binding sites were blocked with 1.0 M ethanolamine-HCl, pH 8.5 for 7 min (10µL/min). Reference surfaces without PR3 were activated and blocked as outlined above for subtraction of non-specific binding and instrument noise. The binding procedure was performed at a flow rate of 5 µL/min at 25 °C in HBS-E. Forty µL of PR3 MAb solutions (0.1 μg/mL and 0.4 μg/mL) were injected at 60 µL/min over the PR3 surface and the dissociation was monitored for 400 s. Each injection was replicated four times for all three analytes at the two different concentrations. All sensor surfaces were regenerated by a 2 min (120 µL) injection of 10 mM HCl between each run. The sensorgrams were fitted to a mathematical model, Langmuir or drifting baseline to calculate binding constants. A standard measure, Chi^2^, was calculated for evaluating the closeness of each fit.

### 2.10. PR3 Ab Sequences

MAb sequences were derived from sequencing cDNA from MAb-producing hybridoma cells (MCLAB, San Francisco, CA, USA).

### 2.11. Amino Acid Analysis (AAA)

Two 4 µg samples of each of the three PR3 preparations (SSI, Arotec and Wieslab) were dried in 500 µL polypropylene vials, hydrolyzed in 6 N HCl and analyzed on a BioChrom 31 amino acid analyzer as described [43]. The best fit was calculated against the composition of PR3, with the addition of zero to seven defensins, and plotted on a curve. The lowest points on the curves were taken as the number of defensins associated with the given PR3 preparation.

### 2.12. Mass Spectrometry (MS)

Approximately 0.5 μg PR3 of each variant was purified using a Poros R1, 50 μm (Applied Biosystems, Carlsbad, CA, USA) microcolumn custom-made in a constricted GELoader tip (Eppendorff, Hamburg, Germany). The column was washed in 100% MeCN and equilibrated with 0.1% TFA prior to sample loading in 20 μL 0.1% TFA. After washing twice with 20 μL 0.1% TFA, the sample was eluted with 0.5 μL 20 mg/mL sinapinic acid in 0.1% TFA, 70% MeCN directly onto a dried droplet of 20 mg/mL sinapinic acid in 100% acetone placed on a metal target. MS was performed in a linear mode on a Bruker ultrafleXtreme MALDI instrument (Bruker Corporation, Billerica, MA, USA).

## 3. Results

### 3.1. Analysis of PR3 Preparations

**Figure 1:**
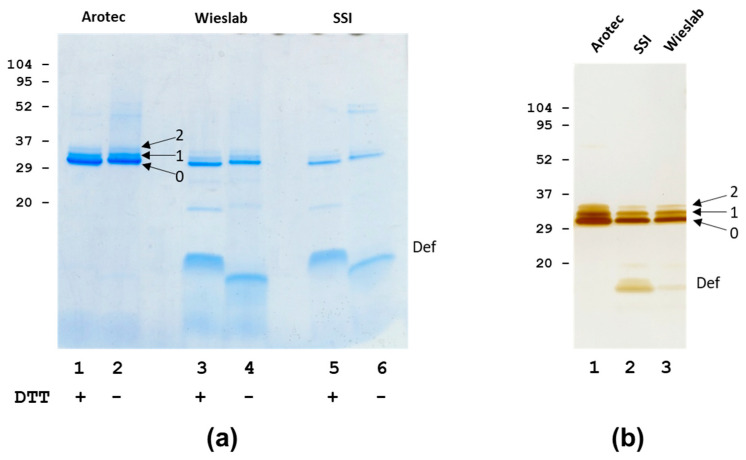
SDS analysis of PR3 preparations. (**a**) Coomassie Brilliant Blue staining. Lanes 1, 2: Arotec PR3, lanes 3, 4: Wieslab PR3, lanes 5, 6: SSI PR3. Samples were boiled with sample buffer with DTT (lanes 1, 3, 5) or without DTT (lanes 2, 4, 6) and run on a 16 % Tricine gel. (**b**) Silver staining. Lane 1: Wieslab PR3, lane 2: SSI PR3, lane 3: Arotec PR3. Samples were boiled with sample buffer with DTT and run on a 4–20% Tris-glycine SDS-PAGE gel. PR3 bands with none, one or two covalently bound defensins are indicated with “0, 1, 2” and free defensins with “Def”.

The analysis of three different PR3 preparations revealed small differences in purity, mainly due to different amounts of associated defensins (Figure 1, Table 1). SDS-PAGE analysis showed that PR3 purified in-house (SSI PR3) had essentially the same composition as PR3 obtained commercially from one company (Wieslab PR3), with a considerable amount of associated defensins. In SDS-PAGE, the PR3, due to its high pI, runs as a somewhat higher Mr band as compared to the exact mass determined by mass spectrometry (Figure 1, Table 1).

In contrast, another commercial preparation (Arotec PR3) had a low content of associated defensins. This difference was observed for several batches of PR3 from the different sources. Despite the differences in associated defensins, PR3 from the three sources generally showed the same pattern of a major PR3 band of Mr of approximately 26 kDa (band 0 or “lower” band), a less intense band above with a Mr of approximately 29 kDa (band 1 or “middle” band) and a weak band of approximately Mr 32 kDa above the middle band (band 2 or “upper” band) (Figure 1, Table 1). Control experiments, which involved boiling Arotec PR3 with an excess of defensins, did not change the relative distribution of the three bands, showing that the upper bands were not an artefact of the sample preparation procedure. From these experiments, it appeared that SSI and Wieslab PR3 contained substantial amounts of defensins released by SDS, whereas Arotec PR3 did not, to a large degree, contain such “loosely” bound defensins. In agreement with this, AAA showed an average of 5–6 defensins per PR3 molecule for SSI and Wieslab PR3, while AAA of Arotec PR3 showed an average of 1–2 defensins per PR3 molecule (Table 1). These findings were confirmed by mass spectrometry, as intact mass analysis of the PR3 preparations showed a pattern of a broad major peak of approximately 25.5 kDa and minor broad peaks of approximately 28.9 kDa and 32.3 kDa for SSI and Wieslab PR3 (Table 1). The major band/peak represented PR3 with different degrees of small glycans, and the upper bands/minor peaks corresponded to the addition of one or two defensins, respectively, as previously described for SSI PR3 [38]. One preparation of Arotec PR3 showed only a major peak of 25.6 kDa in intact mass analysis, indicating very high purity and the absence of the minor species (Table 1).

**Figure 2:**
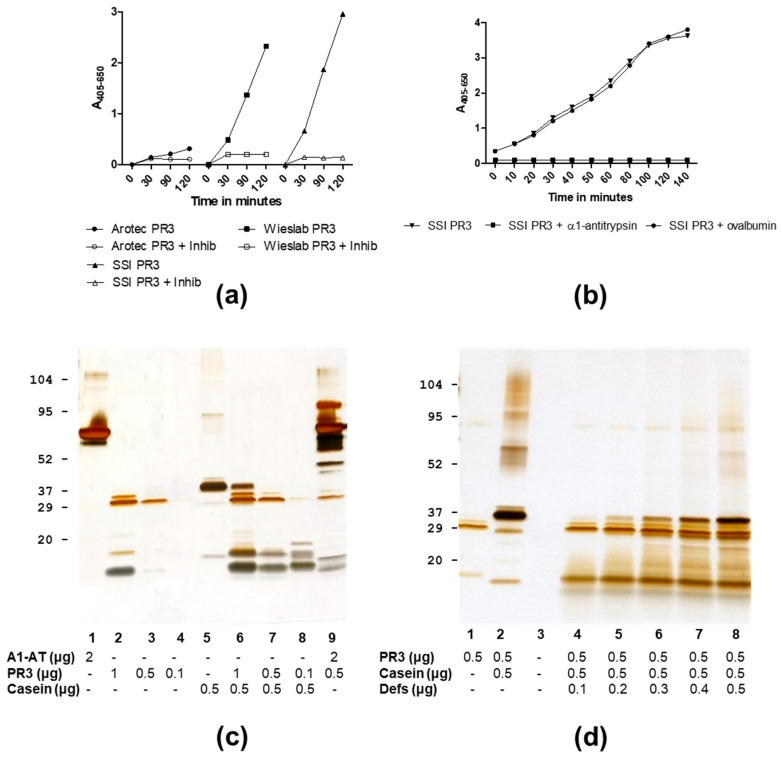
Proteolytic activity of PR3 preparations analysed by competitive inhibition assays and SDS-PAGE followed by silver staining. (**a**) Time-dependent hydrolysis of MeO-Suc-AAPV-PNA by Wieslab, SSI or Arotec PR3 with and without addition of inhibitor (MeO-Suc-AAPV-CMK). (**b**) Hydrolysis of MeO-Suc-AAPV-PNA by SSI PR3 with or without addition of α1-antitrypsin or ovalbumin (4 µg/mL). (**c**) Incubation of casein with varying SSI PR3 amounts pr lane. (**d**) Incubation of casein with SSI PR3 and varying α-defensin amounts pr lane.

PR3 preparations from Wieslab and SSI showed proteolytic activity on the synthetic substrate MeO-Suc-AAPV-pNA and were inhibited by MeO-Suc-AAPV-Cmk. In contrast, Arotec PR3 was essentially inactive or had, at most, a very weak activity (Table 1, Figure 2a). As expected, SSI PR3 proteolytic activity was inhibited by α1-antitrypsin in a concentration-dependent manner, but not by the homologous ovalbumin (Figure 2b). When tested on casein, SSI PR3 surprisingly showed a concentration-dependent activity with less activity at a high PR3 concentration, but pronounced activity at lower PR3 concentrations (Figure 2c). Furthermore, the addition of exogenous defensins inhibited SSI PR3 proteolytic activity towards casein (Figure 2d). Evidently, this pattern of activity was due to the inhibition of proteolytic activity by associated defensins, restricting the access of larger substrates to the active site while allowing access to small substrates.

### 3.2. Characterisation of PR3 MAbs

**Figure 3:**
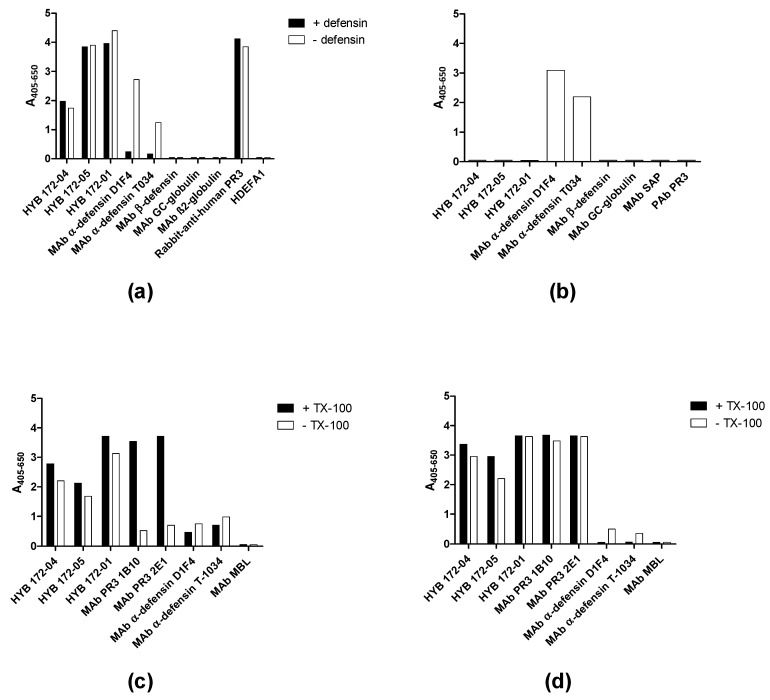
MAb binding to PR3 analysed by ELISA. (**a**) Binding of MAbs to SSI PR3 with or without addition of defensins. (**b**) MAb binding to coated defensins. (**c**) MAb binding to Wieslab PR3 coated with or without Triton X-100 (TX-100). (**d**) MAb binding to Arotec PR3 coated in the absence or presence of Triton X-100.

In light of the unusual glycosylation of PR3 and its association with α-defensins, we undertook a detailed characterisation of the interaction of PR3 with three MAbs (HYB 172-01, HYB 172-04 and HYB 172-05). ELISA showed specific binding to purified SSI PR3 of all three MAbs. The purified SSI PR3 also reacted with MAbs to α-defensin but not with a MAb to β-defensin or other control MAbs (Figure 3a). The binding of defensin MAbs to the coated PR3 could be inhibited by the addition of free defensins, but the binding of the PR3 MAbs was insensitive to the addition of free defensins (Figure 3a), while the PR3 MAbs did not react with coated defensins (Figure 3b).

In the ELISA, it was observed that the three MAbs showed a slightly stronger reaction when SSI PR3 was coated in the presence of 0.01% Triton X-100, a tendency that was even more pronounced for two commercial PR3 MAbs (1B10, 2E1). However, this was less evident when coated with Arotec PR3 containing only a low amount of non-covalently associated defensins (Figure 3c,d), confirming that the MAbs reacted with PR3 itself (or the complex of PR3 and defensins) and not the associated defensins. The three MAbs showed identical titration curves in the ELISA, with 50% of maximal binding at 0.6 µg/mL (Table 2a). Elution assays with different buffers of different pH revealed that HYB 172-04 and 172-05 only dissociated from PR3 at pH = 2, whereas HYB 172-01 dissociated at pH = 4. All three MAbs dissociated at pH = 12, but not at pH = 11 (Table 2).

The MAb PR3 interaction was also investigated by SPR and, due to PR3 requiring detergent for proper solubilisation, these studies were carried out with immobilised PR3. The three PR3 MAbs were found to have strong affinities for the native PR3 complex by SPR, yielding “pseudo-affinity/avidity” constants of 1.8E10 (HYB 172-04), 1.8E11 (HYB 172-05) and 5.2E13 (HYB 172-01) (Table 2b). However, these figures should not be taken as accurate measures of the Abs’ affinities for PR3 (paratope-epitope affinity), but rather only as an indication of the interaction strength.

### 3.3. PR3 Defensin Association

**Figure 4:**
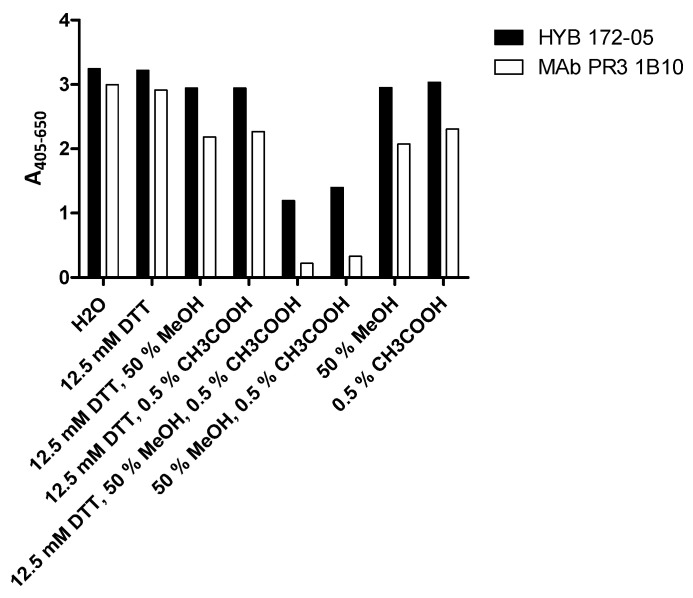
Extraction of coated SSI PR3 and analysis of effect on MAb binding by ELISA.

Defensins associated with PR3 were very resistant to extraction by several agents. However, partial extraction was achieved by a combination of low pH and MeOH (Figure 4).

**Figure 5:**
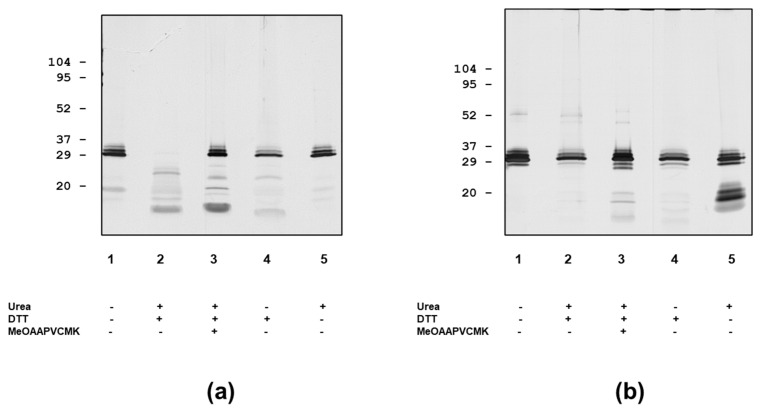
Composition and autoproteolytic activity of PR3 preparations and effect of pre-treatment with urea and DTT as analysed by SDS-PAGE followed by silver staining. (**a**) Wieslab PR3. (**b**) Arotec PR3.

Interestingly, some defensin epitopes became more accessible because of this treatment. The partial release of defensins was also achieved by the sample preparation for SDS-PAGE (Figure 1), but prolonged incubation with a combination of urea and DTT resulted in more extensive defensin extraction (Figure 5). However, the SDS-PAGE analysis showed a considerable degree of autoproteolysis of SSI PR3, even in the high concentrations of urea and DTT (Figure 5). This autoproteolysis was much less for Arotec PR3, which was essentially proteolytically inactive. However, some auto degradation was seen in 8 M urea.

**Figure 6:**
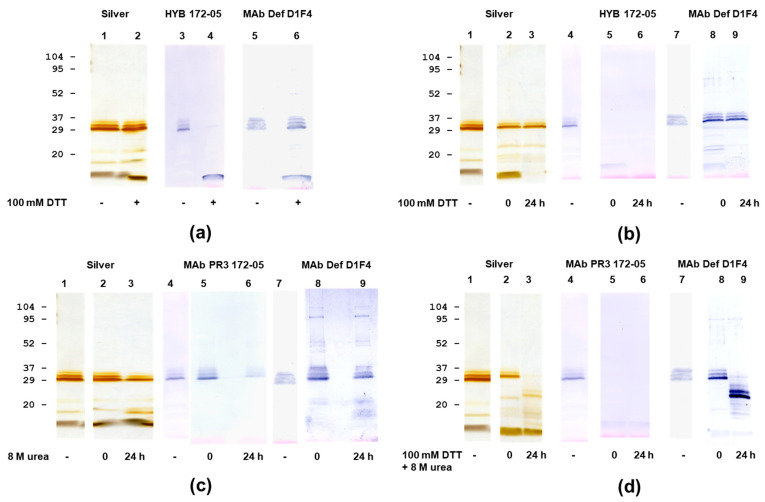
Immunoblotting analysis of PR3 (Wieslab). (**a**). Effect of DTT (100 mM) reduction during sample preparation. (**b**) Effect of long-term high concentration of DTT (100 mM) treatment. (**c**) Effect of long term 8 M urea treatment. (**d**) Effect of long term 8 M urea + 100 mM DTT treatment.

Immunoblotting (Figure 6, Table 2) revealed that the interaction of the three MAbs with PR3 was dependent on intact disulfide bonds. HYB 172-04 and 172-05 reacted with the three characteristic PR3 bands (with 0, 1, 2 covalently bound defensins), also seen by protein staining, whereas HYB 172-01 recognised the two lower bands. These bands were also recognised by MAbs to α-defensins (Figure 6). In the interpretation of the immunoblotting results, it must be remembered that PR3 associates both covalently and strongly/non-covalently with α-defensins. In agreement with this, all PR3 bands reacted with defensin MAbs under both reducing and non-reducing conditions (Figure 6). However, the reaction with defensin MAbs was strongest when using a sample buffer with DTT. Only when using a prolonged dissociation of protein complexes by urea/DTT, followed by SDS PAGE and western blotting, was the reaction of the major PR3 band with defensin MAbs abolished, while the reaction of the minor PR3 bands with α-defensin MAbs was retained (Figure 6).

### 3.4. Inhibition of PR3 MAb Interactions

**Figure 7:**
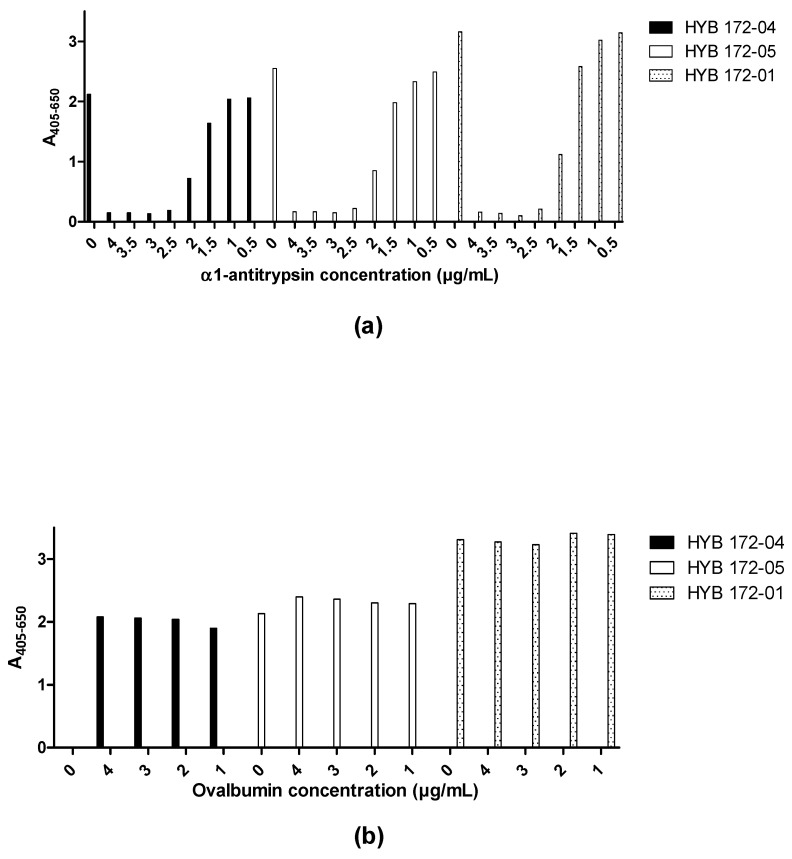
Inhibition by α1-antitrypsin of MAb interaction with PR3 (SSI) analysed by ELISA. (**a**) Inhibition of HYB 172-04, 172-05 and 172-01 with α1-antitrypsin. α1-antitrypsin was pre-incubated in increasing concentrations with PR3 (0.1 µg/well) before coating. (**b**) Control with incubation of HYB 172-04, 172-05 and 172-01 with ovalbumin. Ovalbumin was pre-incubated in increasing concentrations with PR3 (0.1 µg/well) before coating.

None of the MAbs showed inhibition of PR3 proteolytic activity on a small synthetic substrate (MeO-Suc-AAPV-pNA), and the small substrates/inhibitors showed no effects on MAb binding (Table 2). Pre-incubation of PR3 with increasing amounts of α1-antitrypsin before immobilization in a microtitre plate showed complete concentration-dependent inhibition of MAb binding (Figure 7a), whereas no inhibition was seen with the homologous ovalbumin (Figure 7b).

**Figure 8:**
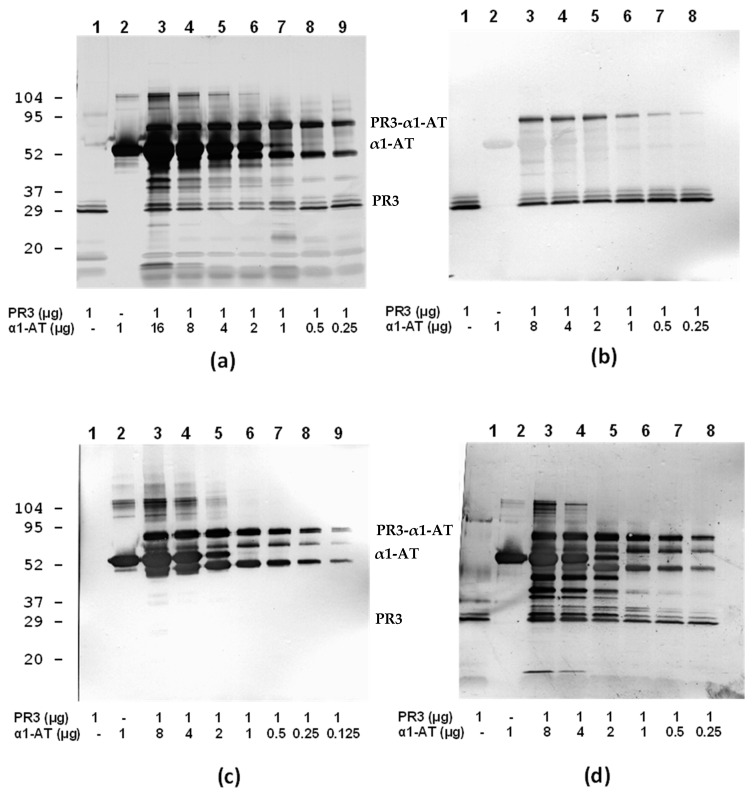
SDS-PAGE and immunoblotting analysis of PR3 (Wieslab) and α1-antitrypsin interactions. (**a**) Silver staining of PR3-α1-antitrypsin complexes at varying amounts. (**b**) Individual and complex staining using HYB 172-05. (**c**) Individual and complex staining using MAb α1-antitrypsin. (**d**) Individual and complex staining using MAb defensin D1F4.

The results also indicated that the PR3 MAbs HYB 172-01, 172-04 and 172-05 bind at the active site of PR3. Analysis of PR3/α-1-antitrypsin complexes using SDS-PAGE and immunoblotting showed a concentration-dependent, irreversible (covalent) interaction with the preservation of MAb binding under non-reducing conditions (Figure 8).

### 3.5. Epitope Mapping with Proteinase 3-Derived Peptides

**Figure 9:**
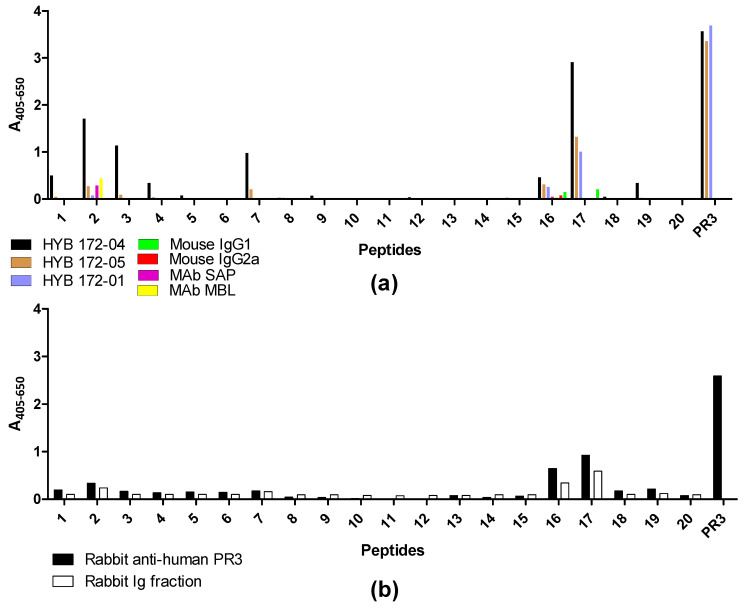
Epitope mapping of PR3 using overlapping peptides coupled to CovaLink-NH microtitre plates and MAb PR3 HYB 172-01, 172-04 and 172-05 analysed by ELISA. PR3 and wells without peptides were used as positive and negative controls, respectively. All wells were corrected for non-specific binding by subtracting reactivity from non-coated wells. (**a**) HYB 172-01, HYB 172-04, HYB 172-05, mouse IgG1 and IgG2a control binding. SAP MAb (IgG1) and MBL MAb (IgG1) binding. SAP Mab and MBL MAb were adjusted to the same concentration as the three PR3 HYBs and used as negative controls. (**b**) Rabbit anti-human PR3 and rabbit IgG binding.

To map the specificity of the PR3 MAbs, we carried out an epitope mapping study with overlapping synthetic peptides covering the PR3 sequence. Every linear peptide contained 20 amino acids with an overlap of 10 amino acids to the following peptide. At the N-terminus of each peptide, a cysteine was introduced in order to allow coupling to activated CovaLink-NH microtitre plates. The reactivity of PR3 MAb HYB 172-01, 172-04, 172-05, irrelevant control MAbs, polyclonal rabbit anti-human PR3 Ab and rabbit immunoglobulins was tested against the immobilized peptides (Figure 9). PR3-specific Abs bound to some extent to several of the peptides, whereas none of the control Abs showed any significant binding. Moderate binding of PR3 MAb HYB 172-04 was seen in peptides 1, 16 and 20, whereas peptides 2, 3, 7 and 17 showed strong binding. PR3 MAb HYB 172-05 showed weak binding to peptides 2, 7 and 16, and moderate binding to peptide 17. PR3 MAb HYB 172-01 showed only weak binding to peptide 16 and moderate binding to peptide 17. The polyclonal rabbit PR3 antiserum essentially exhibited the same binding pattern as that of the monoclonal Abs, although no binding to peptide 7 was seen. These results strongly suggested conformational epitopes.

### 3.6. Monoclonal Antibody Sequences/Modelling

The amino acid sequences of the three Mabs HYB 172-01, 172-04 and 172-05, as well as two additional PR3 Mabs, HYB 172-03 and HYB 206-1, were derived using DNA sequencing (Supplementary S1). Table 3 shows % identity of the amino acid sequences of heavy and light chains of the 5 PR3 Mabs. Most of the similarities were observed for HYB 172-04 and 172-05, both of which had almost identical LCs and 60% identical HCs. HYB 172-01 and HYB 206-01 showed 100% identical LCs and 50% identical HCs and HYB 172-05. Finally, 206-01 showed approximately 60% identical LCs and 90% identical LCs.

### 3.7. Immuno Assays for PR3 Autoantibodies

Routine testing for ANCA using IIF over a period of 4 months revealed 217 positive samples out of 2112 samples. Of the 2112 samples, approximately 2% were cANCA-positive (*n* = 41) and 8% were pANCA-positive (*n* = 176) (Table 4). Of the cANCA-positive samples, approximately 60 % (*n* = 24) were positive in ELISA with in-house purified PR3 and, of the pANCA-positive samples, approximately 40% (*n* = 40) were positive in ELISA with purified MPO. Since PR3 has an unusual glycosylation and associates both covalently and non-covalently with α-defensins, we investigated the possible dependency of the in-house ELISA on the PR3 used.

**Figure 10:**
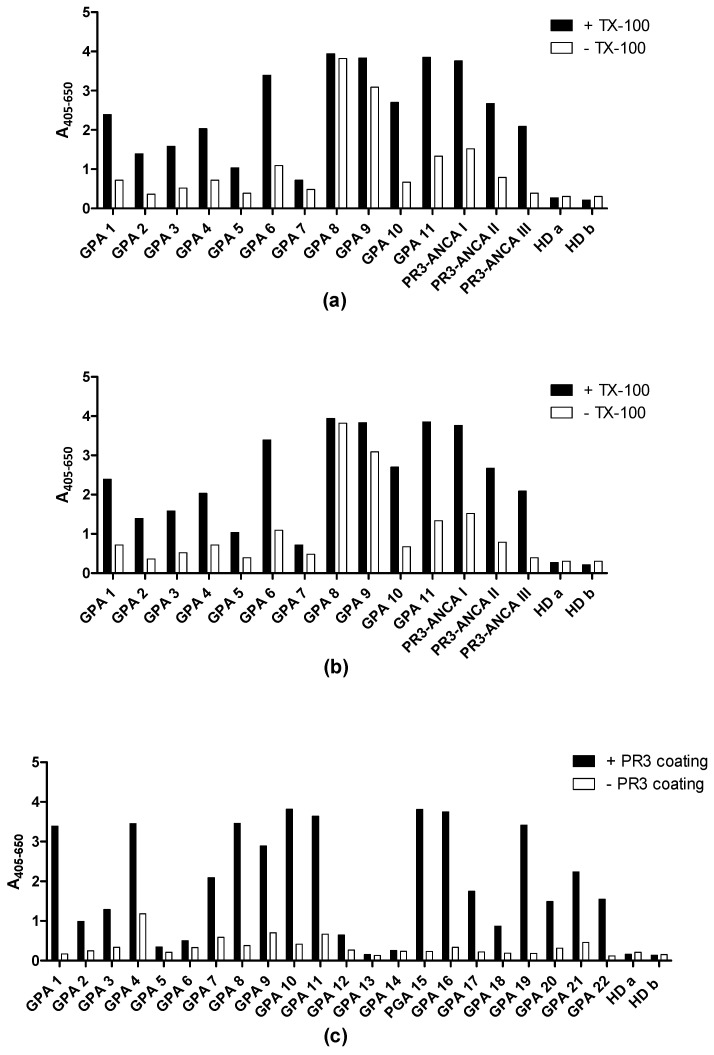
Reactivity of granulomatosis with polyangiitis patient sera (GPA) and healthy controls to PR3 coated in the presence or absence of 0.01% Triton X-100 analysed by ELISA. (**a**) SSI PR3. (**b**) Arotec PR3. (**c**) SSI PR3 without 0.01% Triton X-100 (TX-100).

Coating of plates with PR3 was routinely done with a low amount of detergent (0.01% Triton X-100) in the in-house ELISA. Using both SSI PR3 (which contains both non-covalently and covalently-associated defensins) and Arotec PR3 (which contains essentially only covalently-associated defensins) slightly higher absorbance values were revealed when coating in the presence of Triton X-100, an effect which was most pronounced for Wieslab and SSI PR3 (Figure 10). This result was similar to what was seen with the three Mabs (Figure 3).

**Figure 11:**
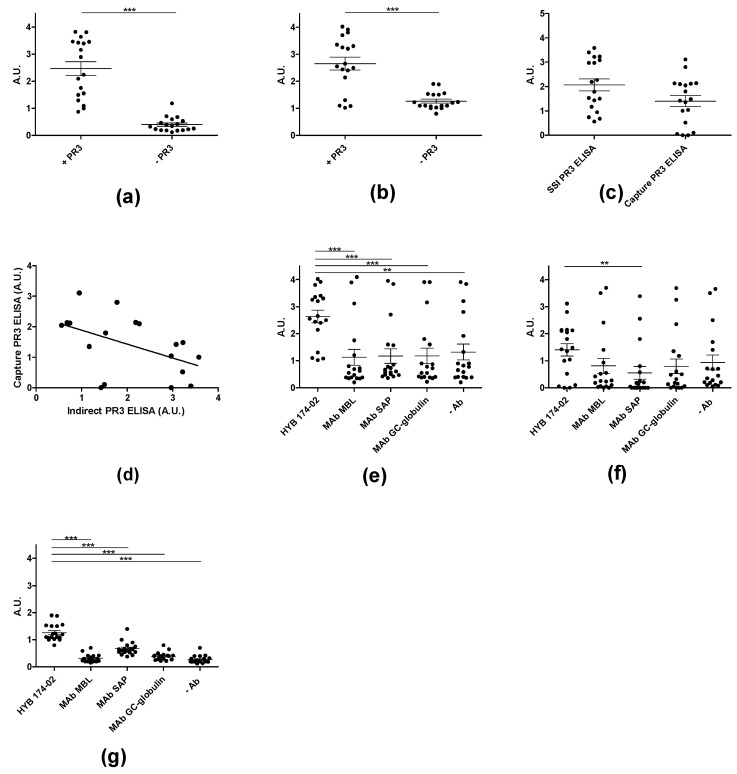
Screening of GPA patient sera binding to PR3 (SSI) by ELISA. (**a**) Detection of autoantibodies in GPA patient sera to PR3 in indirect ELISA, where wells were coated with or without SSI PR3. ** p < 0.01, *** p < 0.001. (**b**) Detection of autoantibodies by capture ELISA using HYB 172-04 as capturing antibody. (**c**) Comparison of indirect (**a**) versus capture ELISA (**b**) for detection of PR3 antibodies. (**d**) Correlation analysis between indirect (**a**) and capture ELISA (**b**). (**e**) PR3 capture ELISA with irrelevant control MAbs. (**f**) Background subtracted PR3 capture ELISA with irrelevant control MAbs. (**g**) PR3 capture ELISA without the presence of PR3 with irrelevant control MAbs.

In the ELISA for PR3 AuAbs, results were routinely corrected for non-specific binding caused by IgG binding to the solid phase. Figure 10a and Figure 11a show ELISA results for patient sera with and without PR3 coating. Many samples showed a relatively high level of non-specific binding. Capture PR3 ELISA revealed similar results as the conventional ELISA (Figure 11b). However, for some samples, the specific signal was close to zero in the capture assay, when results were corrected for background signals (Figure 11c). Surprisingly, the two ELISAs showed a good but negative correlation (Figure 11d), indicating that different effects were operating in the two ELISAs to a significant degree. The simple ELISA can be assumed to measure Abs targeting all epitopes of PR3, including the active site, whereas, in the capture ELISA, the epitopes covered by the capture Abs were inaccessible to patient Abs. Moreover, a relatively high level of non-specific binding was seen for many samples in the capture ELISA (Figure 11e–g). However, the PR3 MAbs exhibited better performance compared to the irrelevant control MAbs, which may be ascribed to the presence of PR3 complexes in the samples. This indicated that the capture effect was, to some extent, mediated by protein–protein interactions of the PR3/defensin complex with the coating Abs and with the solid phase.

**Figure 12:**
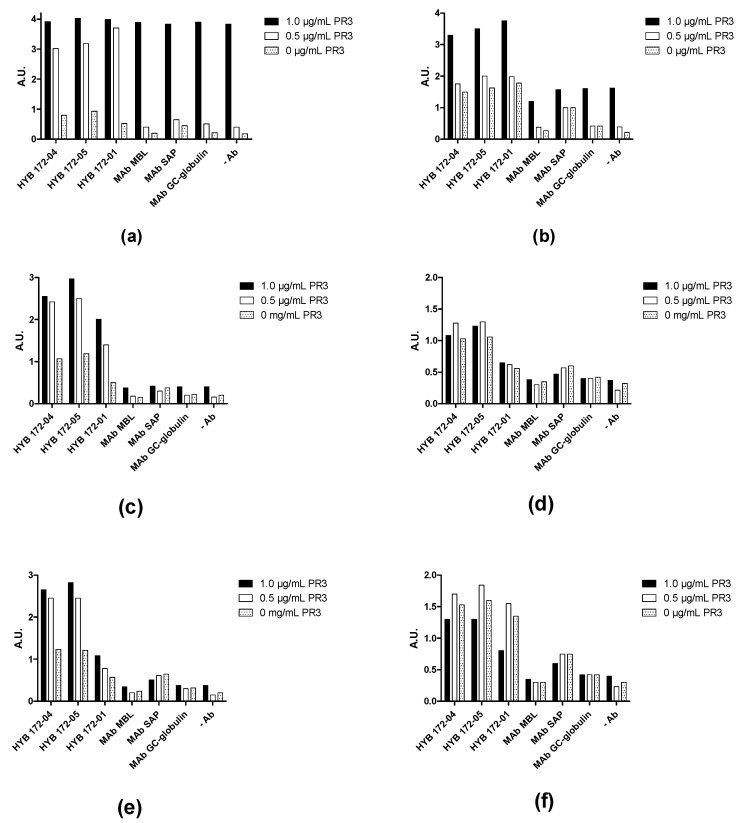
Screening of PR3 autoantibodies in GPA patient sera using PR3 (SSI) concentration-dependent capture ELISA. HYB 172-04, HYB 172-05, HYB 172-01 and irrelevant control MAbs were used as capture antibodies, in combination with PR3 concentrations ranging from 0–1 µg/mL. (**a**–**f**) illustrates the reactivity of 6 GPA PR3 AuAb-positive patients (2 strongly positive (**a**,**b**), 2 middle positive (**c**,**d**) and 2 weakly positive (**e**,**f**)).

This was verified by showing that the effect of PR3 in the capture ELISA was concentration-dependent and varied between different sera (Figure 12). Some sera showed a very high level of non-specific binding at the concentration of PR3 usually used in capture ELISA, independent of the capture Ab (Figure 12a,b), whereas others showed lower levels of non-specific binding (Figure 12c,d) and some showed only non-specific binding (Figure 12e,f).

**Figure 13:**
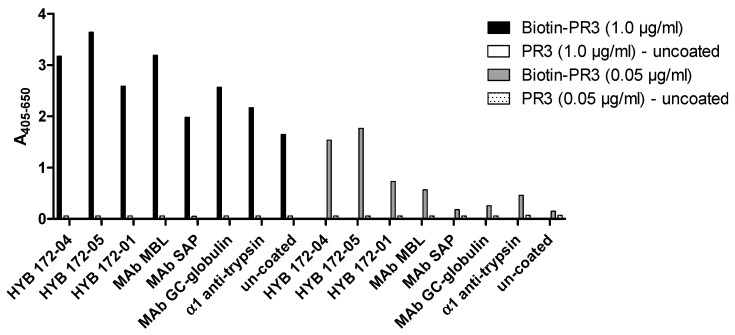
Capture ELISA with coated MAb followed by incubation with biotin-labelled PR3 (SSI) and detection with AP-conjugated streptavidin.

This non-specific interaction of PR3 with Abs was also demonstrated using capture ELISA with coated MAbs, followed by incubation with biotin-labelled PR3 and detection with streptavidin-AP (Figure 13). As seen, the binding was almost equally strong when comparing the PR3 MAbs and irrelevant control MAbs, indicating a high degree of non-specific interactions.

**Figure 14:**
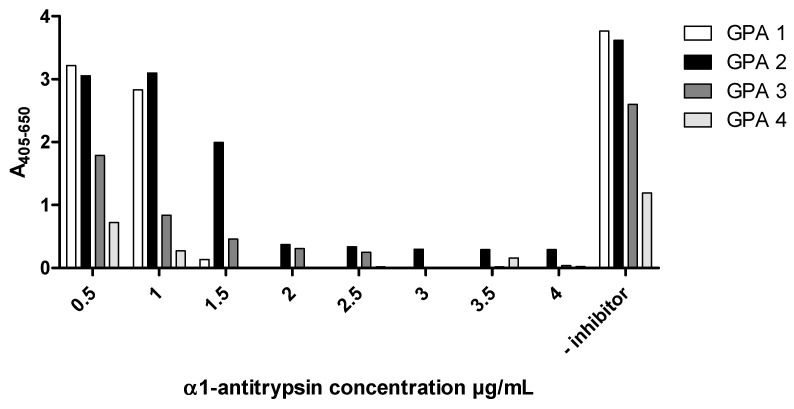
Effect of α1-antitrypsin on autoantibody binding to PR3 (SSI) determined by ELISA. PR3 was pre-incubated with α-1-antitrypsin before coating in ELISA plates followed by incubation with patient sera and detection with AP-conjugated goat-anti human IgG conjugate. GPA 1 and GPA 2 were strong positive for autoantibodies to PR3, whereas GPA 3 was medium positive and GPA 4 was weak positive.

**Figure 15:**
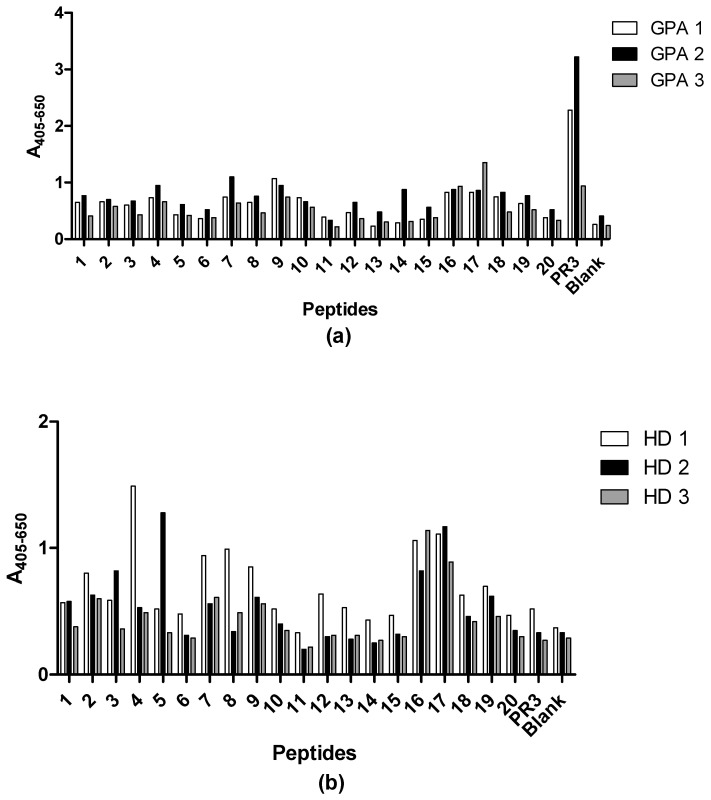
Epitope mapping of PR3 (SSI) using sera from GPA patients and HD and peptide CovaLink-NH ELISA. 20-mer peptides with a 10 amino acid overlap covering the complete proteinase 3 peptide were screened for antibody reactivity. Purified PR3 was used as positive control. (**a**) Reactivity of three GPA patient sera. (**b**) Reactivity of three healthy donor sera.

For all patient samples investigated, complete inhibition of Ab binding to PR3 binding was achieved by pre-incubation of PR3 with α1-antitrypsin (Figure 14), indicating Ab binding at the active site of PR3. Epitope mapping experiments with synthetic peptides did not reveal any significant results, showing that epitopes of patient PR3 Abs are mainly three-dimensional (Figure 15).

## 4. Discussion and Conclusions

The neutrophil granulocyte, which is the most abundant leukocyte in blood, has an important function in the innate immune defense against infections. Its granules harbor multiple antimicrobial agents, including proteases and antimicrobial peptides [1,2]. Among the proteases, PR3 has attracted much interest due to the presence and diagnostic value of PR3 AuAbs in GPA [3,4,5,6,7,8,9]. Additionally, the human neutrophil peptides (HNPs (α-defensins)) have been the subject of much research to elucidate their antimicrobial properties [44,45,46].

**Figure 16:**
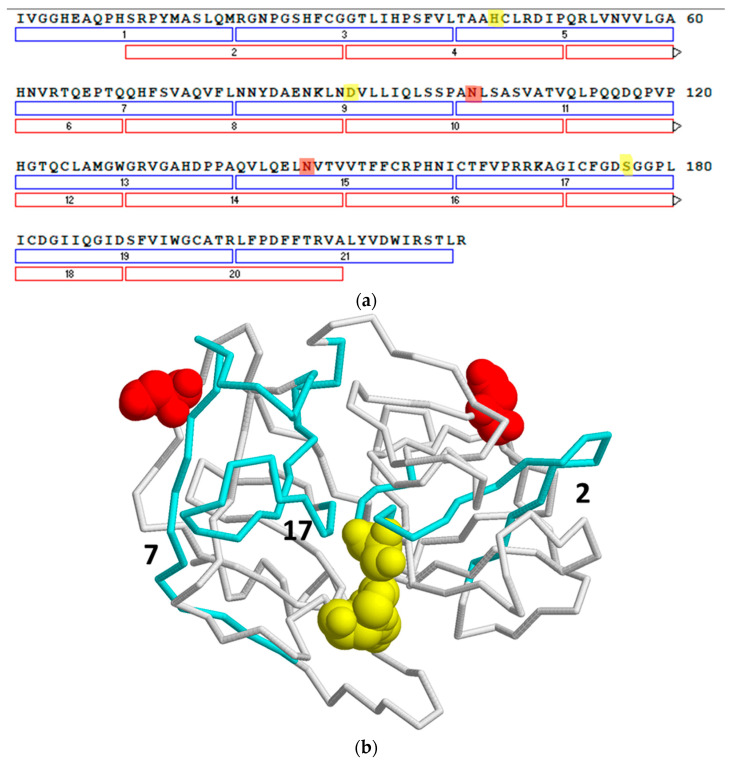
Amino acid sequence and three-dimensional model of human PR3. (**a**). The amino acid sequence of active human PR3 (Uniprot accession number P24158) with the 21 synthesized overlapping peptides indicated with bars and numbers. The three amino acids making up the catalytic triad (His57, Asp102 and Ser195) are shown in a space fill model and marked in yellow, while the two glycosylated asparagine’s are shown in red. (**b**). Three-dimensional structure of PR3 (PDB 1FUJ) shown in backbone view. The catalytic site and glycosylated asparagine’s are shown in space-fill mode and colored like in the sequence. The active catalytic center is in the cleft between the two major domains 1 and 2. By comparing figure (**a**,**b**) it can be seen that peptide 2, 7 and 17 (marked in cyan) are surface-accessible, close in space, and in proximity of the active site.

We have previously characterized the glycosylation of PR3 and shown that it is associated with α-defensins. The glycans of PR3 were found to be severely truncated compared with the fully mature N-linked glycans of MPO, also originating from α-granules [38,47]. The defensins associated with PR3 were shown to consist of both covalently linked and non-covalently associated defensins. However, the nature of the covalent and non-covalent associations and their influence on enzymatic properties and Ab interactions were not characterized in detail. The results presented in this work confirm the covalent and non-covalent association of PR3 with defensins. The covalent association was similar for PR3 from three different sources, and the three preparations generally contained a major band representing PR3 without covalently associated defensins, a “middle” band containing one covalently bound defensin and an “upper” band containing two covalently bound defensin molecules, corresponding to the two N-linked glycosylation sites of PR3 (Figure 16).

In contrast, the degree of non-covalent association with defensins varied among the three PR3 preparations and presumably depended on the method of purification. SSI and Wieslab PR3 contained 5–6 defensin molecules per PR3 molecule, mainly as non-covalently associated defensins, whereas Arotec PR3, presumably purified by a different protocol, had a low amount of non-covalently associated defensins (Table 1). Arotec PR3 was proteolytically inactive, presumably due to chemical inactivation, but SSI and Wieslab PR3 were catalytically active using a small synthetic substrate. However, the non-covalently associated defensins had a concentration-dependent negative impact on the proteolytic activity of PR3 when assayed with casein as substrate. This would seem to provide an explanation for the stability of α-granule proteins against proteolytic (auto)degradation, simply because the defensin concentration is high enough to provide steric hindrance between all the components of the α-granules. In agreement with this, neutrophil elastase has also been found to associate with defensins and to carry small “crippled” N-linked carbohydrates [38,48], indicating a general effect of associated defensins on glycan trimming.

Since PR3 has two surface-exposed lysines, the covalently associated defensins might theoretically be bound through isopeptide bonds between these and the single glutamine of α-defensins. However, no evidence was found for this by mass spectrometry in combination with protease digestion. Instead, the defensins were found to be attached to the glycans residing on Asn102 and 147, most likely by forming Schiff’s bases with the reducing end of the sugar chains.

The strong association between PR3 and defensins provides an explanation for the different behavior of PR3 and MPO upon ethanol fixation and subsequent cytochemistry [49,50,51]. In contrast to MPO, which is solubilized from the granules by this treatment and attaches to the nuclear membrane, giving rise to a perinuclear pattern for MPO AuAbs (pANCA), PR3 remains in the granules, despite its smaller size, and gives rise to a cytoplasmic pattern for PR3 AuAbs (cANCA) because it is trapped in a defensin network.

Both PR3 and defensins have antimicrobial activities [2,11,44,45,46], and defensins have previously been reported to contain lectin-like activity and to insert in and interfere with bacterial membrane integrity [44,45,46,52]. These observations, together with the results presented here, may be particularly relevant for the understanding of PR3-defensin interactions and their role in neutrophil granulocyte anti-microbial activity. The non-covalent and covalent adducts of PR3 with defensins may reflect the surface properties, protease nature of PR3 and the general binding and lectin-like properties of defensins. Both non-covalently and/or covalently bound defensins may target PR3 to pathogen membranes and cell walls, where the protease may degrade essential targets. Thus, the PR3/defensin complexes may have a synergistic antimicrobial activity by virtue of PR3s and the defensins’ individual antimicrobial activities.

All three PR3 preparations were capable of binding α1-antitrypsin, which abolishes the proteolytic activity of serine proteases by binding irreversibly to the active site [53,54,55,56]. This process ends with minor conformational changes in the active catalytic center of PR3 and loss of activity in a stable covalent acyl-enzyme complex with α1-antitrypsin. Inhibition of PR3 MAb binding by α1-antitrypsin demonstrated that the MAbs bind at the active site. Ovalbumin was used as a control protein, as it has sequence and structural homology with other members of the serpin superfamily, as well as a molecular size (45 kDa) similar to that of α1-antitrypsin (52 kDa) [56,57,58]. These results agree with other reports, although some sera showed a reaction with the PR3-α1AT complex, whereas others reported no binding of most patient Abs to the PR3-α1AT complex. Some Abs interfered with PR3 complexation with α1-AT [7,8,17,29,59,60]. Here, peptide epitope mapping results also indicated the binding of the MAbs at the active site. However, there were some differences, as MAb HYB 172-04 and HYB 172-05, but not HYB 172-01, bound strongly to peptide 17 (Figure 3).

In immunoblotting, two of the MAbs, HYB 172-04 and HYB 172-05 recognized all three PR3 bands, while HYB 172-01 only bound the two lower bands. Furthermore, they barely reacted with PR3 separated by reducing SDS-PAGE. In agreement with this, we found that the MAbs bind at closely located, partly overlapping sites, encompassing peptide 17, a sequence from the substrate binding site of PR3. By highlighting the peptides’ interaction with the PR3-specific Abs in an X-ray crystallography model of PR3 [14], it is clear that peptide 17 and, to some extent, peptide 2 are located in the vicinity of the active site at Serine 176, while peptide 7 is located at a greater distance to the active site (Figure 16). This is in agreement with previous studies, which also found peptide epitopes located near the active site [32,33]. Furthermore, all three peptides are easily accessible at the surface of the PR3. Altogether, our results indicate that the MAbs depend on the intact three-dimensional structure of the PR3-defensin complex for optimal binding and that they bind at the active site, possibly reflecting the stability of the protease-defensin Ab complex, when the active site is blocked in a non-competitive inhibitory way.

Abs, which bind at the active site of PR3 and are inhibited by α1-AT, may theoretically inhibit the proteolytic activity of the enzyme. Some studies have shown that PR3 Abs inhibit the enzymatic activity of PR3, especially when using a large substrate (elastin, casein), whereas others have found that PR3 enzymatic activity is not influenced by the Abs [7,27,38,59,60,61].

Since results with patient-derived PR3 Abs generally recapitulated those with the MAbs, showing binding to three-dimensional epitopes at the active site of PR3 and inhibition by α-1-antitrypsin, this would theoretically predict that capture assays for PR3 AuAbs based on these MAbs would not work. Yet, MAb PR3 capture assays have been reported to complement direct coating ELISAs and to have similar or even improved sensitivity compared with these [36,62,63,64,65]. The results presented here indicate that the capture effect of some (PR3) MAbs is partly due to a direct protein–protein interaction between PR3 and the coating MAbs, possibly mediated by the PR3-associated α-defensins, which are known to interact with a variety of different molecules [44,45,46]. Our results may also explain why it has been difficult to define PR3 AuAb epitopes using different methods, simply because the epitopes depend, to varying degrees, on PR3/defensin associations. For example, the three MAbs used here (Table 2) were, in previous work, assigned to clearly different epitopes, denoted by 1, 3 and 4 [27], whereas here, they seemed to overlap considerably. Thus, there are conflicting results in the literature regarding epitope specificity and assay characteristics, etc. in relation to PR3 which can now at least partly be explained by the use of different PR3 preparations with differences in defensin associations, use of different MAbs and different substrates.

Finally, the current results may have implications for consensus recommendations for ANCA testing, especially PR3 autoantibody immunoassays [66]. In fact, the recommendation to use mixtures of different PR3 preparations in such assays may directly reflect the differences in defensin associations of PR3 prepared by different protocols [67]. In any case, the implications of the current results for the design of PR3 immunoassays and for understanding the etiology of ANCA-associated vasculitis deserve further study.

In conclusion, we have mapped the epitopes of several PR3 MAbs and patient-derived PR3 AuAbs to the active site and showed that the epitopes of these Abs depend on the three-dimensional structure of the PR3/defensin complex.

## Figures and Tables

**Table 1:** Characteristics of PR3 preparations.

Preparation	Mr/SDS-PAGE	Mw/MS	Defensins ^a^	Activity ^b^	Inhibition ^c^	α1-AT Binding ^d^
SSI	26, 29, 32	25.5, 29.0, 32.3	5–6	+	+/+/+	+
Wieslab	26, 29, 32	25.5, 28.9, 32.4	5–6	+	+/+/+	+
Arotec	26, 29, 32	25.6, −, −	1–2	−	NA	+

^a^ No of associated defensins as determined by amino acid analysis. ^b^ Proteolytic activity with MeO-Suc-AAPV-PNA. ^c^ MeO-Suc-AAPV-CMK/PMSF/ α1-AT. ^d^ Determined by ELISA and/or SDS-PAGE. NA: not applicable. ND: not determined.

**Table 2:** Characteristics of PR3 MAbs. (**a**) Basic parameters. (**b**). SPR results. Kinetic binding parameters of MAbs interaction with immobilised SSI PR3 based on mathematical fitting to Langmuir and drifting base-line models.

**(a)**						
**PR3 MAb**	**Type**	**50% Binding** **(µg/mL)**	**Elution pH** **(Low/High)**	**Inhibition of ** Binding *	**α1-AT ** **Inhibition**	**Immuno Blotting ^#^**
HYB 172-01	IgG1,κ (6A6)	0.6	4/12	−/−/−	+	26, 29
HYB 172-04	IgG2a,κ (4A3)	0.6	2/12	−/−/−	+	26, 29, 32
HYB 172-05	IgG2a,κ (4A5)	0.6	2/12	−/−/−	+	26, 29, 32
**(b)**						
**PR3 Mab**	**k_a_ (1/Ms)**	**k_d_ (1/s)**	**K_a_ (1/M)**		**K_d_ (M)**	**Chi^2^**
HYB 172-01	2.72 × 10^6^	5.21 × 10^−8^	5.23 × 10^13^		1.91 × 10^−14^	4.03
HYB 172-04	1.03 × 10^7^	5.71 × 10^−4^	1.8 × 10^10^		5.55 × 10^−11^	0.152
HYB 172-05	1.24 × 10^7^	6.84 × 10^−5^	1.81 × 10^11^		1.81× 10^11^	4.8

* Inhibition by MeO-Suc-AAPV-PNA, MeO-Suc-AAPV-CMK, PMSF ^#^ Reactivity with bands of Mr.

**Table 3:** Sequence similarity matrix for proteinase 3 monoclonal antibodies. (**a**). Heavy chains. (**b**). Light chains.

**(a)**					
**HYB**	**172-05**	**206-1**	**172-03**	**172-01**	**172-04**
172-05	100.00	92.66	63.55	60.38	60.38
206-1	92.66	100.00	65.42	61.32	61.32
172-03	63.55	65.42	100.00	55.77	55.77
172-01	60.38	61.32	55.77	100.00	100.00
172-04	60.38	61.32	55.77	100.00	100.00
**(b)**					
**HYB**	**172-04**	**172-05**	**172-03**	**206-1**	**172-01**
172-04	100.00	98.28	62.93	57.76	48.28
172-05	98.28	100.00	64.66	59.48	48.28
172-03	62.93	64.66	100.00	64.66	45.30
206-1	57.76	59.48	64.66	100.00	50.86
172-01	48.28	48.28	45.30	50.86	100.00

**Table 4:** ANCA, PR3 and MPO Abs in patient serum samples tested by IIF and ELISA. 2112 samples were tested in IIF and 217 ANCA-positive samples (C-ANCA and (P-ANCA) were tested in ELISA. C-ANCA positive samples were tested for PR3 Abs, while P-ANCA positive samples were tested for MPO Abs.

Indirect Immunofluorescence Microscopy			Enzyme-Linked Immunosorbent Assay			
*N* = 2112			*N* = 41		*N* = 176	
Antibody	C-ANCA Positive	P-ANCA Positive	PR3 Positive	PR3 Negative	MPO Positive	MPO Negative
Samples	41	176	24	17	70	106
%	2	8	59	41	40	60

## Data Availability

All related data and methods are presented in this paper. Additional inquiries should be addressed to the corresponding author.

## Supplementary Materials

The following supporting information can be downloaded at: https://www.mdpi.com/article/10.3390/antib12010023/s1, Supplementary S1: PR3 MAb sequences.
