# Supplementary material for: Defensin Interactions in Relation to Monoclonal and Disease-Related Proteinase 3 Antibodies Binding at the Catalytic Site
Source: Antibodies (Basel). 2023 Mar 13;12(1):23. doi: 10.3390/antib12010023 (PMC10044823; doi:10.3390/antib12010023)
Supplement: Supplementary file 1 [file antibodies-12-00023-s001.zip › antibodies-2122293-supplementary.pdf]

Supplementary S1. PR3 MAb sequences

Red letters: leader sequence

Green letters: Variable region sequence.

Bold , underline and Italic: indicate 3 CDR regions

172-01

Antibody Heavy Chain

>DNA level

AAGCAGTGGTATCAACGCAGTANTACGCGGGGGACAGAGGAGGCCGGTCCTGGATTCGATTCCCAGTTCCTCACATT
CAGTCAGCACTGAACACGGACCCCTCACC***ATG***AACTTCGGGCTCAGCTTGATTTTCCTTGCCCTCATTTTAAAAGGTGT
CCAGTGTGAGGTGCAGCTGGAGCAGTCAGGGGGAGACTTAGTGAAGCCTGGAGGGTCCCTGAAACTCTCCTGTGCA
GCCTCTGGATTCACTTTCAGTACCTATGGCATGTCTTGGGTTCGCCAGACTCCAGACAAGAGGCTGGAGTGGGTCGCA
ACCTTTGTTAGTGGTGGTAGTTATAACTACTATCCAGACAGTGTGAAGGGGCGATTCGCCATTTCCAGAGACAATGCC
AAGAACGCCCTGTACCTGCAAATGAGCAGTCTGAAGTCTGAGGACACAGCCATGTATTATTGTGTAAGACGGGGGC
TTCCTACGATGCTCTGGACTACTGGGGTCAAGGAACCTCAGTCACCGTCTCCTCAGCCAAAACGACACCCCCATCTGTC
TATCCACTGGCCCCTGGATCTGCTGCCCAAACTAACTCCATGGTGACCCTGGGATGCCTGGTCAAGGGCTATTTCCCTG
AGCCAGTGACAGTGACCTGGAACTCTGGATCCCTGTCCAGCGGTGTGCACACCTTCCCAGCTGTCCTGCAGTCTGACC
TCTACACTCTGAGCAGCTCAGTGACTGTCCCCTCCAGCACCTGGCCCAGCGAGACCGTCACCTGCAACGTTGCCCACCC
GGCCAGCAGCACCAAGGTGGACAAGAAAATTGTGCCCAGGGATTGTGGTTGTAAGCCTTGCATATGTACAGTCCCAG
AAGTATCATCTGTCTTCATCTTCCCCCCAAAGCCCAAGGATGTGCTCACCATTACTCTGACTCCTAAGGTCACGTGTGTT
GTGGTAGACATCAGCAAGGATGATCCCGAGGTCCAGTTCAGCTGGTTTGTAGATGATGTGGAGGTGCACACAGCTCA
GACGCAACCCCGGGAGGAGCAGTTCAACAGCACTTTCCGCTCAGTCAGTGAACTTCCCATCATGCACCAGGACTGGCT
CAATGGCAAGGAGTTCAAATGCAGGGTCAACAGTGCAGCTTTCCCTGCCCCCATCGAGAAAACCATCTCCAAAACCAA
AGGCAGACCGAAGGCTCCACAGGTGTACACCATTCCACCTCCCAAGGAGCAGATGGCCAAGGATAAAGTCAGTCTGA
CCTGCATGATAACAGACTTCTTCCCTGAAGACATTACTGTGGAGTGGCAGTGGAATGGGCAGCCAGCGGAGAACTAC
AAGAACACTCAGCCCATCATGGACACAGATGGCTCTTACTTCGTCTACAGCAAGCTCAATGTGCAGAAGAGCAACTGG
GAGGCAGGAAATACTTTCACCTGCTCTGTGTTACATGAGGGCCTGCACAACCACCATACTGAGAAGAGCCTCTCCCAC
TCTCCTGGTAAATGATCCCAGTGTCCTTGGAGCCCTCTGGTCCTACAGGACTCTGACACCTACCTCCACCCCTCCCTGTA
TAAATAAAGCACCCAGCACTGCCTTGGGACCCTGCAAAAAAAAAAAAAAAAAAAATTTGCGAGGACGGTG

>Amino acid level

MNFGLSLIFLALILKGVQCEVQLEQSGGDLVKPGGSLKLSCAASGFTFSTYGMSWVRQTPDKRLEW
VATFVSGGSYNYYPDSVKGRFAISRDNAKNALYLQMSSLKSEDTAMYYCVRRGASYDALDYWGQ
GTSVTVSSAKTTPPSVYPLAPGSAAQTNSMVTLGCLVKGYFPEPVTVTWNSGSLSSGVHTFPAVLQS
DLYTLSSSVTVPSSTWPSETVTCNVAHPASSTKVDKKIVPRDCGCKPCICTVPEVSSVFIFPPKPKDVL
TITLTPKVTCVVVDISKDDPEVQFSWFVDDVEVHTAQTQPREEQFNSTFRSVSELPIMHQDWLNGKE
FKCRVNSAAFPAPIEKTISKTKGRPKAPQVYTIPPPKEQMAKDKVSLTCMITDFFPEDITVEWQWNG
QPAENYKNTQPIMDTDGSYFVYSKLNVQKSNWEAGNTFTCSVLHEGLHNHHTEKSLSHSPGK

172-01

Antibody light Chain

>DNA level

AAGCAGTGGTATCAACGCAGAGTACGCGGGGGAAATACATCAGGCAGGCAAGGGCATCAAG**ATG**AAGTCACAGAC
CCAGGTCTTCGTATTTCTACTGCTCTGTGTGTCTGGTGCTCATGGGAGTATTGTGATGACCCAGACTCCCAAATTCCTG
CTTGTATCAGCAGGAGACAGGATTACCATAACCTGCAAGGCCAGTCAGAGTGTGAGTAATGATGTAGCTTGGTACCA
ACAGAAGCCAGGGCAGTCTCCTAAACTGCTGATATACTATGCATCCAATCGCTACACTGGAGTCCCTGATCGCTTCACT
GGCAGTGGATATGGGACGGATTTCACTTTCACCATCAGCACTGTGCAGGCTGAAGACCTGGCAGTTTATTTCTGTCAG
CAGGATTATAGCTCTCCATTCACGTTCGGCTCGGGGACAAAGTTGGAAATAAAACGGGCTGATGCTGCACCAACTGTA
TCCATCTTCCCACCATCCAGTGAGCAGTTA

>Amino acid level

MKSQTQVFVFLLLCVSGAHGSIVMTQTPKFLLVSAGDRITITC***KASQSVSNDVA***WYQQKPGQSPKLLIY***YASNR
YT***GVPDRFTGSGYGTDFTFTISTVQAEDLAVYFC***QQDYSSPFT***FGSGTKLEIKRA

172-03

Antibody Heavy Chain

>DNA level

AAGCAGTGGTATCAACGCAGAGTACGCGGGACACACAGAAAAACATGAGATCACAGTTCTCTCTACAGTCACTGAAC
ACACAGGACCTCACC**ATG**GGATGGAGCTATATCATCCTCTTTTTGGTAGCAACAGTTACAGATGTCCACTCCCAGGTC
CAGTTGCAGCAGGCTGGGGCCGAGCTTGTGAAGCCTGGGGCTTCAGTGAAGTTGTCCTGCAAGGCTTCTGGCTACAC
CTTCACCAACTACTGGATGCACTGGGTGAAGCAGAGGCCTGGACAAGGCCTTGAGTGGATTGGAGAGATTAATCCCA
CCAACGGTGGTATTAATTACAATGAGGAGTTCATAAAGAAGGCCACACTGACTGCAGACAAATCGTCCAACACAGCCT
ACATGCAGCTCAGCGGCCTGACATCTGAGGACTCTGCGGTCTATTTCTGTTTAATTGAGATTATGACGGGGGAGGGGT
ACTGGGGCCAAGGCACTTCTCTCACAGTCTCCTCAGCCAAAACGACACCCCCATCTGTCTATCCACTGGCCCCTGGATC
TGCTGCCCAAACTAACTCCATGGTGACCCTGGGATGCCTGGTCAAGGGCTATTTCCCTGAGCCAGTGACAGTGACCTG
GAACTCTGGATCCCTGTCCAGCGGTGTGCACACCTTCC

>Amino acid level

MGWSYIILFLVATVTDVHSQVQLQQAGAELVKPGASVKLSCKAS***GYTFTNY***WMHWVKQRPGQGLEWIGEI***N
PTNGG***INYNEEFIKKATLTADKSSNTAYMQLSGLTSEDSAVYFCLI***EIMTGEGY***WGQGTSLTVSSAKTTPPSVYPL
APGSAAQTNSMVTLGCLVKGYFPEPVTVTWNSGSLSS

172-03

Antibody light Chain

>DNA level

AAGCAGTGGTATCAACGCAGAGTACGCGGGGGAGGCAGGGGGAGCAAGATGGATTCACAGGCCCAGGTCCTC**ATG**
TTGCTGCTGCTATCGGTATCTGGTACTTGTGGAGACATTTTGATGACCCAGTCTCCATCCTCCCTGACTGTGTCAGCGG
GAGAGAAGGTCACTATGAGTTGTAAGTCCAGTCAGAATCTCTTAGTTAGTGGCAACCAAAATAACTACTTGGCCTGGC
ACCAGCAGAAACCAGGACGATCTCCTAAAATGCTGATAATTTGGGCATCCACTAGGGTGTCTGGAGTCCCTGATCGCT
TCATAGGCAGTGGATCTGGGACGGATTTCACTCTGACCATCAACAGTGTGCAGGCTGAAGATCTGGCTGTTTATTACT
GTCAGCAGTCCTTCAGCGCTCCGCTCACGTTCGGTGCTGGGACCAAGCTGGAGCTGAAACGGGCTGATGCTCCACCA
ACTGTATCCACC

>Amino acid level

MDSQAQVLMLLLLSVSGTCGDILMTQSPSSLTVSAGEKVTMSCKSS***QNLLVSGNQNNY***LAWHQQKPGRSPK
MLII***WAS***TRVSGVPDRFIGSGSGTDFTLTINSVQAEDLAVYYC***QQSFSAP***LTFGAGTKLELK

172-04 GR13033PCS (Note: the 5' leader sequencing still missing)

Antibody Heavy Chain

>DNA level

**CTTCCGGAGGTGAAGCTGGAGCAGTCTGGACCTGACCTGGTGAAGCCTGGGGCTTCAGTGAAGATATCCTGCAAG
GCTTCTGGTTACTCTTTCACTGGCTACTACCTGCACTGGGTGAAACAGAGCCATGGAAAGAGCCTTGAGTGGATTGG
ACGTGTCAATCCTAACAATGATATTACTACCTACAACCAGAAGTTCAAGGACAAGGCCATATTAACTGTAGACAAGT
CATCCAGCACAGCCTACATGGAGTTCCGCAGCCTGACATCTGAGGACTCTGCGGTCTATTACTGTGCAGGGGGGGG
TAATGCTTTGGACTACTGGGGTCAAGGAACCTCAGTCACCGTCTCCTCAGCCAAAACAACAGCCCCATCGGTCTATC
CACTGGCCCCTGTGTGTGGAGATACAACTGGCTCCTCGGTGACTCTAGGATGCCTGGTCAAGGGTTATTTCCCTGAG
CCAGTGACCTTGACCTGGAACTCTGGATCCCTGTCCAGTGGTGTGCACACCTTCCCAGCTGTCCTGCAGTCTGACCTC
TACACCCTCAGCAGCTCAGTGACTGTAACCTCGAGCACCTGGCCCAGCCAGTCCATCACCTGCAATGTGGCCCACCC
GGCAAGCAGCACCAAGGTGGACAAGAAAATTGAGCCCAGAGGGCCCACAATCAAGCCCTGTCCTCCATGCAAATG
CCCAGCACCTAACCTCTTGGGTGGACCATCCGTCTTCATCTTCCCTCCAAAGATCAAGGATGTACTCATGATCTCCCTG
AGCCCCATAGTCACATGTGTGGTGGTGGATGTGAGCGAGGATGACCCAGATGTCCAGATCAGCTGGTTTGTGAACA
ACGTGGAAGTACACACAGCTCAGACACAAACCCATAGAGAGGATTACAACAGTACTCTCCGGGTGGTCAGTGCCCT
CCCCATCCAGCACCAGGACTGGATGAGTGGCAAGGAGTTCAAATGCAAGGTCAACAACAAAGACCTCCCAGCGCCC
ATCGAGAGAACCATCTCAAAACCCAAAGGGTCAGTAAGAGCTCCACAGGTATATGTTTTGCCTCCACCAGAAGAAG
AGATGACTAAGAAACAGGTCACTTTGACCTGCATGGTCACAGACTTCATGCCTGAAGACATTTACGTGGAGTGGAC
CAACAACGGGAAAACAGAGCTAAACTACAAGAACACTGAACCAGTCCTGGACTTTGATGGTTTTTACTTCATGTACA
GCAAGCTGAGAGTGGAAAAGAAGAACTGGGTGGAAAGAAATAGCTACTCCTGTTCAGTGGTCCACGAGGGTTTGC
ACAATCACCACACGACTAAGAGCTTTTCCCGGACTCCGGGTAAATGAGCTCAGCACCCACAAAACTTTCAGGTCCAA
AGAGACACCCCCACTCATCTCCATGCTTCCCTTGTATAAATAAAGCACCCAGCAATGCCTGGGACCATGTAAAAAAA
AAAAAAAAAAAAAAATTTGCGAGGACGGTG**

>Amino acid level

EVKLEQSGPDLVKPGASVKISCKASGYSFTGYYLHWVKQSHGKSLEWIGRVNPNNDITTYNQKFKDKAILTVDKS
SSTAYMEFRSLTSEDSAVYYCAGGGNALDYWGQGTSVTVSSAKTTAPSVYPLAPVCGDTTGSSVTLGCLVKGYF
PEPVTLTWNSGSLSSGVHTFPAVLQSDLYTLSSSVTVTSSTWPSQSITCNVAHPASSTKVDKKIEPRGPTIKPCPPC
KCPAPNLLGGPSVFIFPPKIKDVLMISLSPIVTCVVVDVSEDDPDVQISWFVNNVEVHTAQTQTHREDYNSTLRV

VSALPIQHQDWMSGKEFKCKVNNKDLPAPIERTISKPKGSVRAPQVYVLPPPEEEMTKKQVTLTCMVTDFMPE
DIYVEWTNNGKTELNYKNTEPVLDFDGFYFMYSKLRVEKKNWVERNSYSCSVVHEGLHNHHTTKSFSRTPGK

172-04 GR13033PCS

Antibody Light Chain

>DNA level

GGTGGATACAGTTGGTGGAGCATCCTCTCTTCCAGCTCTCAGAG**ATG**GAGACAGACACACTCCTGTTATGGGTACTG
CTGCTCTGGGTTCCAGGTTCCACTGGTGACATTGTGCTGACACAGTCTCCCAAATTCCTGCTTGTATCAGCAGGAGACA
GGGTTACCATAACCTGCAAGGCCAGTCAGAGTGTGAGTAATAATGTAGGTTGGTACCAACAGAAGACAGGACAGTCT
CCTAAACTGCTGATATACTACGCATCCAATCGATACACTGGAGTCCCTGATCGCTTCACTGGCAGTGGATATGGGACG
GATTTCACTTTCACCATCAGCACTGTGCAGGCTGAAGACCTGGCAGTTTATTTCTGTCAGCAGGATTATAACTCTCCGT
ACACGTTCGGAGGGGGGACCAAGTTGGAAATAAAACGGGCTGATGCTGCACCAACTGTATCCATCTTCCCACCATCCA
GTGAGCAGTTAACATCTGGAGGTGCCTCAGTCGTGTGCTTCTTGAACAACTTCTACCCCAAAGACATCAATGTCAAGT
GGAAGATTGATGGCAGTGAACGACAAAATGGCGTCCTGAACAGTTGGACTGATCAGGACAGCAAAGACAGCACCTA
CAGCATGAGCAGCACCCTCACGTTGACCAAGGACGAGTATGAACGACATAACAGCTATACCTGTGAGGCCACTCACA
AGACATCAACTTCACCCATTGTCAAGAGCTTCAACAGGAATGAGTGTTAGAGACAAAGGTCCTGAGACGCCACCACCA
GCTCCCCAGCTCCATCCTATCTTCCCTTCTAAGGTCTTGGAGGCTTCCCCACAAGCGACCTACCACTGTTGCGGTGCTCC
AAACCTCCTCCCCACCTCCTTTTCCTCCTCCTCCCTTTCCTTGGCTTTTATCATGCTAATATTTGCAGAAAATATTCAATAA
AGTGAGTCTTTGCACTTGAAAAAAAAAAAAAAAAAAAAATTTGCGAGGACGGTG

>Amino acid level

METDTLLLWVLLXWVPGSTGEIVLTQSPAIMSASLGEEITLTCSASSSVSYMHWYQQKSGTSPKLL
IYSTSNLASGVPSRFSGSGSGTFYSLTISSVEAEDAADYYCHQWSSYGTFGGGTKLEIKRADAAPT
VSIFPPSSEQLTSGGASVVCFLNNFYPKDINVKWKIDGSERQNGVLNSWTDQDSKDSTYSMSSTLT
LTKDEYERHNSYTCEATHKTSTSPIVKSFNRNEC

172-05

Antibody Heavy Chain

>DNA level

AAGCAGTGGTATCAACGCAGAGTACGCGGGGACCACAGCCCCTGAAGACACTGACTCTAACT**ATG**GAATGGAGCTG
GGTCTTTCTCTTCCTCCTGTCAGTAACTACAGGTGTCCACTCTGAGGTCCAGCTGCAGCAGTCTGGACCTGACCTGGTG
AAGCCTGGGGCTTCAGTGAAGATATCCTGCAAGGCTTCTGGTTACTCTTTCACTGGCTACTACCTGCACTGGGTGAAA
CAGAGCCATGGAAAGAGCCTTGAGTGGATTGGACGTGTCAATCCTAACAATGATATTACTACCTACAACCAGAAGTTC
AAGGACAAGGCCATATTAACTGTAGACAAGTCATCCAGCACAGCCTACATGGAGTTCCGCAGCCTGACATCTGAGGA
CTCTGCGGTCTATTACTGTGCAGGGGGGGGTAATGCTTTGGACTACTGGGGTCAAGGAACCTCAGTCACCGTCTCCTC
AGCCAAAACAACAGCCCCATCGGTCTATCCACTGGCCCCTGTGTGTGGAGATACAACTGGCTCCTCGGTGACTCTAGG

ATGCCTGGTCAAGGGTTATTTCCCTGAGCCAGTGACCTTGACCTGGAACTCTGGATCCCTGTCCAGCGGTGTGCACACCTTCC

>Amino acid level

MEWSWVFLFLLSVTTGVHSEVQLQQSGPDLVKPGASVKISCKAS***GYSFTGYY***LHWVKQSHGKSLEWIGR***VNPNNDIT***TYNQKFKDKAILTVDKSSSTAYMEFRSLTSEDSAVYYC***A***GGGNALDYWGQGTSVTVSS

172-05

Antibody light Chain

>DNA level

AAGCAGTGGTATCAACGCAGAGTACGCGGGAGAAATACATCAGGCAGGCAAGGGCATCAAG**ATG**AAGTCACAGACCCAGGTCTTCGTATTTCTACTGCTCTGTGTGTCTGGTGCTCATGGGAGGATTGTGATGACCCAGACTCCCAAATTCCTGCTTGTATCAGCAGGAGACAGGGTTACCATAACCTGCAAGGCCAGTCAGAGTGTGAGTAATAATGTAGGTTGGTACCAACAGAAGACAGGACAGTCTCCTAAACTGCTGATATACTACGCATCCAATCGATACACTGGAGTCCCTGATCGCTTCACTGGCAGTGGATATGGGACGGATTTCACTTTCACCATCAGCACTGTGCAGGCTGAAGACCTGGCAGTTTATTTCTGTCAGCAGGATTATAACTCTCCGTACACGTTCGGAGGGGGGACCAAGTTGGAAATAAAACGGGCTGATGCTGCACCAACTGTATCCATCTTCCCACCATCCAGTGAGCAGTTA

>Amino acid level

MKSQTQVFVFLLLCVSGAHGRIVMTQTPKFLLVSAGDRVTITC***KASQSVSNNVG***WYQQKTGQSPKLLIY***YASNRYT***GVPDRFTGSGYGTDFTFTISTVQAEDLAVYFC***QQDYNSPYT***FGGGTKLEIKRA

206-1

Antibody Heavy Chain

>DNA level

AAGCAGTGGTATCAACGCAGAGTACGCGGGGATATGAGCCCTATCTTCTCTACAGACACTGAATCTCAAGGTCCTTACA**ATG**CAATGCAGCTGGGTTATCTTCTTCCTGATGGCAGTGGTTACAGGGGTCAATTCAGAGGTTCAGCTGCAGCAGTCTGGGGCAGAGGTTGTGAAGCCAGGGGCCTCAGTCAAGTTGTCCTGCACAGCTTCTGGCTTCAACATTAAAGACACCTATATGTACTGGGTGAAGCAGAGGCCTGAACAGGGCCTGGAGTGGATTGGAAGGATTGATCCTGCGAATGGTAATACTAAATATGACCCGAAGTTCCAGGGCAAGGCCACTATAACAACAGACACATCCTCCAACACAGCCTACCTGCAGCTCAGCAGCCTGACATCTGAGGACACTGCCGTCTATTACTGTGCTAGACGGAGTAGCGGTTTTGACTGCTGGGGCCAAGGCACCACTCTCACAGTCTCCTCAGCCAAAACGACACCCCCATCTGTCTATCCACTGGCCCCTGGATCTGCTGCCCAAACTAACTCCATGGTGACCCTGGGATGCCTGGTCAAGGGCTATTTCCCTGAGCCAGTGACAGTGACCTGGAACTCTGGATCCCTGTCCAGCGGTGTGCACACCTTCC

>Amino acid level

MQCSWVIFFLMAVVTGVNSEVQLQQSGAEVVKPGASVKLSCTAS***GFNIKDT***YMYWVKQRPEQGLEWIGRI***DP***
ANGNTKYDPKFQGKATITTDTSSNTAYLQLSSLTSEDTAVYYCAR***RSSGFDC***WGQGTTLTVSS

206-1

Antibody light Chain

>DNA level

AAGCAGTGGTATCAACGCAGAGTACGCGGGGGAAATACATCAGGCAGGCAAGGGCATCAAG**ATG**AAGTCACAGAC
CCAGGTCTTCGTATTTCTACTGCTCTGTGTGTCTGGTGCTCATGGGAGTATTGTGATGACCCAGACTCCCAAATTCCTG
CTTGTATCAGCAGGAGACAGGATTACCATAACCTGCAAGGCCAGTCAGAGTGTGAGTAATGATGTAGCTTGGTACCA
ACAGAAGCCAGGGCAGTCTCCTAAACTGCTGATATACTATGCATCCAATCGCTACACTGGAGTCCCTGATCGCTTCACT
GGCAGTGGATATGGGACGGATTTCACTTTCACCATCAGCACTGTGCAGGCTGAAGACCTGGCAGTTTATTTCTGTCAG
CAGGATTATAGCTCTCCATTCACGTTCGGCTCGGGGACAAAGTTGGAAATAAAACGGGCTGATGCTGCACCAACTGTA
TCCATCTTCCCACCATCCAGTGAGCAGTTA

>Amino acid level

MKSQTQVFVFLLLCVSGAHGSIVMTQTPKFLLVSAGDRITITC***KASQSVSNDVA***WYQQKPGQSPKLLIY***YASNR***
YTGVPDRFTGSGYGTDFTFTISTVQAEDLAVYFC***QQDYSSPFT***FGSGTKLEIKRA
